# Working memory training and evaluation based on brain-computer interface and virtual reality: our opinion

**DOI:** 10.3389/fnhum.2023.1291983

**Published:** 2023-10-24

**Authors:** Dingna Duan, Zhonglin Wu, Yanhong Zhou, Xianglong Wan, Dong Wen

**Affiliations:** ^1^State Key Laboratory of Cognitive Neuroscience and Learning, Beijing Normal University, Beijing, China; ^2^IDG/McGovern Institute for Brain Research, Beijing Normal University, Beijing, China; ^3^School of Information Science and Engineering, Yanshan University, Qinhuangdao, China; ^4^School of Mathematics and Information Science and Technology, Hebei Normal University of Science and Technology, Qinhuangdao, China; ^5^School of Intelligence Science and Technology, University of Science and Technology Beijing, Beijing, China

**Keywords:** working memory evaluation, working memory training, brain-computer interface, virtual reality, electroencephalography

## 1. Introduction

Working memory (WM) is the cognitive system necessary for the temporary storage and manipulation of information during complex tasks like reasoning, comprehension, and learning (Baddeley, 1992, 2010). Studies have shown that the WM capacity improves in response to working memory training (WMT) through purposeful guidance and targeted cognitive training (Shipstead et al., 2012; Melby-Lervåg and Hulme, 2013; Finc et al., 2020). How to conduct effective WM training and evaluation is a crucial research topic. With the development of science and technology, the combination of brain-computer interface (BCI) and virtual reality (VR) technology, called BCI-VR, is an emerging technology with great potential for effective WMT. The BCI-VR system combines the immersive sensory feedback of VR with real-time electroencephalography (EEG) signals of brain activity, making cognitive training more engaging and efficient compared to traditional methods (Elbamby et al., 2018; Wen et al., 2021). In recent years, BCI-VR has been widely used in the field of rehabilitation medicine for a variety of diseases, including autism (Amaral et al., 2017), stroke (Lechner et al., 2014; Vourvopoulos and Bermúdez I Badia, 2016), attention deficit hyperactivity disorder (ADHD) (Rohani and Puthusserypady, 2015), Parkinson's disease (Morales-Gomez et al., 2018). However, the neuroimaging studies on BCI-VR in WM training and evaluation are still in its early stages, and more work needs to be done to ultimately effectively improve the WM capacity.

In this opinion article, we will analyze the relevant literature on current WM training and EEG signal analysis methods for WM evaluation, illustrate the value of BCI-VR and its application in WMT, and discuss the current challenges, as well as potential future directions. It is expected that these analyses will shed light on the field of WMT.

## 2. Current landscape and challenges

### 2.1. WM training based on VR

WM constitutes the core for executing various cognitive tasks, encompassing attention control, reasoning, and daily learning activities, underscoring its pivotal role in maintaining normal cognitive function. Existing research has highlighted that task-based WMT can progressively enhance WM capacity while also positively impacting broader cognitive skills and learning abilities (Vartanian et al., 2013; Gathercole et al., 2019; Sala et al., 2019). With an evolving focus on innovative WMT approaches, VR technology has been introduced into digital games. With its highly immersive environment and natural interaction, it has gradually become a research hotspot in the field of cognitive training. Notably, VR-based WMT has shown promise, with studies revealing its ability to significantly enhance sustained attention in real-life classroom environments (Coleman et al., 2019). Additionally, it has demonstrated efficacy in improving event-based prospective memory deficits in patients with major depressive disorder (MDD), suggesting its potential as a therapeutic intervention (Huang et al., 2022). The Nesplora Aquarium VR test, designed for assessing attention and WM in adults, has garnered attention for its robust psychometric properties and reliability, offering a valuable tool for evaluating attentional capacity in adults (Climent et al., 2021). Furthermore, investigations into the influence of valence and arousal on WM performance during VR gaming have yielded intriguing results, indicating that playing in VR led to improved WM performance, particularly in individuals with lower WM capacity (Gabana et al., 2017). This suggests a link between affective states and WM performance in VR gaming (Gabana et al., 2017). This convergence of research suggests the promising potential of VR-based WMT, offering an exciting avenue for advancing cognitive training paradigms beyond traditional methods.

### 2.2. WM evaluation based on BCI

BCI connects the brain to the external environment, allowing visualization of neural changes in the brain during cognitive activities through EEG signals. How to effectively conduct the EEG signal analysis to objectively describe the cognitive activities of the brain and accurately evaluate the effect of cognitive training is a crucial topic in WMT.

#### 2.2.1. Frequency-based EEG signal analysis

Studies have shown that analyzing EEG signals in specific frequency bands, can reflect changes in cognitive activities. For example, the features of EEG signals in the Alpha and Beta frequency bands, extracted through the power spectral density method, could reflect the changes in cognitive performance during noise exposure (Jafari et al., 2019). The spatial features of EEG signals in the Gamma frequency band, extracted through the co-space algorithm, could reflect the changes in different emotional performances (Li and Lu, 2009). Theta-band EEG signal fluctuations are associated with learning and memory activities (Herweg et al., 2020). As for the studies on WM, researchers investigated the neural mechanism of WM activity and examine neural changes associated with WM based on EEG signals. Herweg et al. have explored the performance of the brain when it achieves successful memory by studying the Theta-band EEG signal fluctuations, revealing that successful memory is related to both a broad-band tilt of the power spectrum and enhanced narrow-band Theta oscillations (Herweg et al., 2020). Additionally, studies have investigated the key role of “active-silent” neural states in WM using a perturbation method that can measure mnemonic hidden states in EEG, revealing that memory-item-specific information can be decoded from impulse responses, and that the accuracy of WM-guided behavior (including memory precision) could be predicted by the strength of hidden-state coding (Wolff et al., 2017).

#### 2.2.2. Connectivity-based EEG signal analysis

It is also a significant scientific topic to study WM from the perspective of coupling relationships between cortical regions. Notably, it has been reported that mutual information can analyze the coupling relationship of multi-channel EEG signals. Guevara et al. (2018) reported significant differences in the mutual information of the Theta frequency band in cognitive activities. Li and Ouyang (2010) introduced a permutation conditional mutual information method to effectively estimate the directional coupling between neuronal populations that are mutually interconnected. Additionally, Buriro et al. (2018) utilized different types of inter-channel relationships in EEG as feature sets for continuous prediction of microsleep states. Toppi et al. (2018) discovered that different topological properties of EEG-derived networks, analyzed using graph-theoretic analysis, can reveal complex underlying WM phases, such as a higher small-world topology during the encoding phase of WM. More effective EEG signal analysis methods are needed for better evaluation of WMT.

### 2.3. Current challenges

Nowadays, EEG neurofeedback technology has been applied to WMT in many studies, and VR technology has also been initially introduced into WMT. However, the application of the integration of EEG and VR in WM training and evaluation remains limited and is in its early developmental stage. In addition, the methods used to evaluate the effects of WMT in current studies are still mainly limited to neuropsychological scale assessments, the accuracy of users' responses to cognitive tasks, and the subjective judgment of researchers. These methods may not be able to accurately evaluate the WM capacity, nor provide timely scientific evidence on whether the cognitive training process achieves effective enhancement in WM capacity. Hence, it is essential to integrate emerging technologies like VR with objective and quantitative evaluation methods like EEG signal analysis into WMT, to enhance the effectiveness of cognitive task training and evaluation.

### 2.4. The status of BCI-VR technology and its application in WM training and evaluation

#### 2.4.1. The research value and status of BCI-VR

VR technology can simulate immersive virtual reality scenes and provide users with visual, auditory, and other sensory feedback, which makes the cognitive training more attractive and engaging. EEG- can provide the direct interaction between brain and the external devices, which can intuitively and real-time display the neural changes in subjects' brain activity during training, and can effectively evaluate subjects' WM, which is much more objective and quantitative than traditional neuropsychological scale assessment (Elbamby et al., 2018; Wen et al., 2021). Compared with other BCI technologies like functional magnetic resonance imaging (fMRI), near-infrared spectroscopy (NIRS), and magnetoencephalography (MEG), EEG provides a more cost-effective, portable, and real-time method for monitoring brain activity. Therefore, EEG-based BCI-VR might be a promising way for WM training and evaluation.

In recent years, BCI-VR has been widely used in rehabilitation medicine for various diseases. Recent studies have explored the combination of EEG and electromyography (EMG) with VR for facial palsy and consciousness disorders rehabilitation, demonstrating improved outcomes (Qidwai et al., 2019; Maggio et al., 2020). A novel P300-based BCI paradigm utilizing VR and social cues shows potential for training joint-attention skills in autism (Amaral et al., 2017). Additionally, multimodal VR simulations combined with motor priming hold promise for stroke neurorehabilitation (Vourvopoulos and Bermúdez I Badia, 2016). A portable BCI-VR tool for ADHD has shown initial promise (Rohani and Puthusserypady, 2015). Besides, VR treatment has shown effective effects on Parkinson's disease (Morales-Gomez et al., 2018).

#### 2.4.2. BCI-VR in WM training and evaluation

Research on BCI-VR in WM training and evaluation is still in its early stages, and a few recent studies have preliminarily investigated its effectiveness. One study adopted a quantitative approach, utilizing EEG signals and game performance data to assess cognitive levels (Wan et al., 2021). The findings revealed that VR games outperformed 3D counterparts in enhancing WM and attention, leading to notably shorter task completion times and faster attention peaks, underscoring the significant potential of VR for advanced cognitive training (Wan et al., 2021). Another investigation examined VR's impact on visual working memory, finding minor effects on accuracy and reaction time without statistical significance, and EEG data suggested perceptual-level cognitive changes over task performance alterations (Redlinger et al., 2022). Additionally, a study explored a cognitively adaptive VR training system incorporating real-time EEG to adjust task difficulty, revealing significant brain activity changes with increasing complexity while response times remained unchanged (Dey et al., 2019). Furthermore, a study explored the integration of VR headsets with EEG in cognitive tasks, confirming that VR head-mounted displays (HMDs) could successfully elicit ERP components similar to traditional computer screens, indicating their potential utility in early ERP investigations for cognitive research (Aksoy et al., 2021). These studies collectively demonstrate the evolving landscape of WMT, underscored by the promising prospects of BCI-VR, offering more immersive and quantifiable approaches to cognitive enhancement.

### 2.5. Future directions of WM training and evaluation based on BCI-VR

For the future direction of WMT, the combination of BCI and VR technology, which can provide immersive training environments to attract the subjects to focus on training, and collect EEG signals in real-time for cognitive training evaluation, is a promising option for conducting effective WMT. In WM training, more interaction ways, such as gestures or utilizing EEG signals, can replace the manual operation in traditional digital games to control virtual characters in VR games, thus enhancing the training experience and improving the effectiveness of WMT. In addition, in WM evaluation, in terms of EEG signal analysis, multiple feature extraction methods can be used for feature coupling analysis, thus further verifying the analysis from multiple perspectives. Moreover, for WM evaluation, relying solely on EEG signals and behavioral data may not provide a comprehensive assessment. Subsequently, NIRS, fMRI, and various physiological signals such as eye movement and electrocardiogram signals, can be fused to evaluate the WMT effect from multiple perspectives. Furthermore, for the BCI-VR system, it is important to further improve its software and hardware integration to make the device more portable and low-cost, and to make WM training and evaluation more efficient and accurate.

## 3. Conclusions

In conclusion, this study analyzed the current research status of WM training and evaluation, and emphasizing challenges and future directions related to BCI-VR technology. The integration of novel VR technology into WMT, known for its immersive environment and natural interaction, has gained prominence in cognitive training research. Additionally, the real-time visualization of neural changes through EEG signals positions BCI as a valuable tool for quantitatively and objectively assessing the effects of WMT. In consequence, our opinion is that the BCI-VR technology combined with EEG signal analysis is of great potential in WM training and evaluation, and can effectively help improve individual cognitive capabilities and performance. Currently, research in BCI-VR-based WMT is mainly in the experimental phase, but with technological advancements, it will hold substantial potential to benefit diverse populations across education, healthcare, mental health, aging, and sports domains, bearing significant socioeconomic value.

## Author contributions

DD: Funding acquisition, Writing—original draft. ZW: Writing—original draft, Investigation. YZ: Investigation, Writing—original draft. XW: Investigation, Writing—original draft. DW: Funding acquisition, Methodology, Supervision, Writing—review and editing.
